# Role of Serum Markers in Combination as a Diagnostic Tool for Acute Pulmonary Embolism: Cross-Sectional Study

**DOI:** 10.7759/cureus.10584

**Published:** 2020-09-22

**Authors:** Muhammad Yousuf, Sara Reza, Saleha Zafar, Shehnaz Noor, Lubna Sarfraz, Muhammad Iqbal, Talha Laique

**Affiliations:** 1 Pathology, Quaid-e-Azam Medical College, Bahawalpur, PAK; 2 Cardiology, Cardiac Centre, Bahawalpur, PAK; 3 Pharmacology, Lahore Medical and Dental College, Lahore, PAK

**Keywords:** acute pulmonary embolism, serum markers

## Abstract

Background and objective

Acute pulmonary embolism (APE) is a serious cardiovascular emergency, mainly secondary to deep vein thrombosis (DVT), which causes death. The goal of the current study was to determine the levels of different serum markers in combination among patients with and without acute pulmonary embolism in order to use them as a diagnostic tool.

Methodology

A sample of 96 patients was kept with a 90% power of study and a 5% level of significance in the current study. It was carried from January to June 2020 in the Department of Medicine, Bahawal Victoria Hospital, Bahawalpur, after the hospital’s Ethical Committee approval. Written informed consent was taken. Serum levels of C-reactive protein (CRP), D-dimer, fibrinogen, and Troponin-I between both groups were done once enrolled. SPSS software, version 25 (IBM Corp. Armonk, NY) was used to analyze the collected data.

Results

Patients with acute pulmonary embolism had a mean age of 50.4 ± 10.4 years. All serum markers were significantly raised in patients suffering from acute pulmonary embolism with a p-value of <0.05.

Conclusion

We concluded that all these serum markers can be used together as a tool in making the correct diagnosis of acute pulmonary embolism in our setup.

## Introduction

Acute pulmonary embolism (APE) is a serious health emergency, mainly secondary to deep vein thrombosis (DVT), which causes death, especially in patients with poor hemodynamic status and comorbidities [1-2]. Its signs and symptoms are very vague and misleading to make a correct diagnosis [3]. The first-line diagnostic investigation for pulmonary embolism (PE) is computed tomographic (CT) angiography. Although, it has few contraindications that include renal insufficiency, cost-effectiveness, and pregnancy among patients, especially in developing countries like Pakistan. Hence, efforts were made to find some low-priced and less-invasive investigations for its correct diagnosis. Many serum biochemical markers like troponin-I, fibrinogen, and C-reactive protein (CRP) independently have established their prognostic role in these patients [4].

D-dimer is a degradation product of cross-linked fibrin that increases in acute thromboembolic events [5]. In addition, high levels of biochemical markers, such as plasma fibrinogen and Troponin-I, predict adverse events in acute pulmonary embolism [6]. However, these serum markers have a nonspecific diagnostic and prognostic value when used alone, as they get raised in many other conditions that include old age, malignancies, hemodynamic impairment, infections, cardiac, lung diseases, and postoperative states as an acute-phase reactant [7]. Many of these conditions have signs and symptoms similar to those of APE [8].

The immediate initiation of thrombolytic therapy is critical in the management of this emergency condition in order to reduce patient mortality and morbidity [9]. In light of this increasing burden, we planned the current study to determine the levels of different serum markers when used in combination as a diagnostic tool among patients with and without acute pulmonary embolism.

## Materials and methods

A sample of 96 patients (48 per group) was kept with a 90% power of study and a 5% level of significance in the current study. It was carried from January to June 2020 in the Department of Medicine, Bahawal Victoria Hospital, Bahawalpur. It was a cross-sectional comparative study. Only patients fulfilling the inclusion criteria, i.e. both genders (age 20-60 years), with signs and symptoms of acute pulmonary embolism, were enrolled throughout the project following approval from the hospital’s ethical committee. Written informed consent was taken. Serum levels of CRP, D-dimer, fibrinogen, and Troponin-I between both groups were done once enrolled. Those who failed to give consent or fulfill the inclusion criteria and had any malignancy or any other serious disease like fibrosis, myocardial infarction (MI), or pulmonary tuberculosis were ruled out [8].

## Results

In the present study, the majority of enrolled subjects were females, and their baseline characteristics are depicted in Table 1:

**Table 1:** Baseline characteristics distribution among enrolled patients APE: acute pulmonary embolism

Variables	Categories	Without APE	With APE	p-value
Gender	Male	22 (45.8%)	16 (33.3%)	0.211
	Female	26 (54.2%)	32 (66.7%)	
Age	≤ 45 years	25 (52.1%)	18 (27.5%)	0.151
	> 45 years	23 (47.9%)	30 (62.5%)	
Mean age ± S.D (years)		44.9 ± 11.2	50.4 ± 10.4	0.614
Mean CRP ± S.D (mg/l)		12.4 ± 5.8	22.4 ± 8.2	0.019*

a comparison of different serum biomarkers was made among both groups. Significant p-values depicted their positive association with the disease, as given in Table 2.

**Table 2:** Comparison of serum biomarkers between patients with and without APE APE: acute pulmonary embolism

Variables	Without APE	With APE	p-value
D-dimer (μg/ml)	2.58 ± 1.44	4.45 ± 2.46	< 0.001*
Fibrinogen (mg/dl)	485.2 ±78.3	463.3 ±112.6	0.014*
Troponin-I (ng/mL)	0.24 ± 0.21	0.55 ± 0.38	< 0.001*

## Discussion

Although many previous studies have shown an association between individual hemostatic markers and risk of acute pulmonary embolism, the use of such markers in patients with acute chest pain in combination as a diagnostic tool remained unclear. In developing countries where the economy is an issue, expensive investigations cannot be routinely done; hence, the present study was conducted. It was an attempt to make simple investigations a powerful diagnostic tool.

Acute pulmonary embolism is the third most common cause of cardiovascular-related deaths. It accounts for 10% among hospitalized patients, with a correct diagnosis before death accounting for only 29% [10]. It is a hypercoagulable state. The literature review revealed that many serum markers of coagulation and inflammation increase in acute pulmonary embolism but when assessed in combination as a diagnostic tool, this remained unclear [11].

Fibrinogen is a soluble plasma glycoprotein that is converted into fibrin during blood coagulation states [12]. It increases in acute conditions as an acute-phase reactant. A previous study revealed that fibrinogen levels in APE patients (498 ± 369 mg/dl) were similar to those without APE (520 ± 268 mg/dl), with insignificant p = 0.29.23 [13].These results were paradoxical to our results, which showed a significant difference between fibrinogen levels in both groups.

Raised levels of D-dimers usually indicate thrombus formation that can terminate as vigorous plaques, as revealed by many previous studies. Similarly, our findings showed that raised D-dimer levels were present among patients suffering from acute pulmonary embolism having significant p-value (<0.001*) [1]. Hence, our results were in conformity with previous findings.

Patients can develop acute pulmonary embolism without having raised cardiac enzymes like Troponin-I, as shown previously [14]. Paradoxically, other studies showed that levels of Troponin-I were high among patients suffering from pulmonary embolism [15]. Similarly, in the current project, our results showed that high levels of Troponin-I among patients suffering acute pulmonary embolism were present with a significant p-value of <0.001*.

It was reported that the marker of inflammation (CRP) was raised in patients suffering from thrombus-forming states as well as inflammatory disorders. Similarly, our results were in line with previous works and showed a significant difference in CRP levels between both groups with a p-value of <0.019* [12].

The findings of the present study depicted that all serum markers like fibrinogen, D-dimers, troponin-I, and CRP were significantly raised in patients suffering from acute pulmonary embolism. All of them can be investigated together in patients suffering from acute chest pain, thus helping in making the correct diagnosis of acute pulmonary embolism in the majority of the cases in developing countries, although investigations like computed tomographic (CT) angiography cannot be replaced due to its correct diagnosis. Our findings were in the same direction as other investigators.

Limitations

It was a single-center study with small sample size. The time constraint and the handful of resources were other limitations.

## Conclusions

There were significantly higher levels of serum markers like D-dimers, fibrinogen, Troponin-I, and CRP among patients with acute pulmonary embolism in the present study. The differences between both groups (with and without acute pulmonary embolism) in terms of serum markers were significant. Therefore, all these serum markers can be used together as a tool for making the correct diagnosis of acute pulmonary embolism in our setup.
